# Demystifying Surgical Training in the UK: A Trainee-Led Global Teaching Initiative for Surgical Career Aspirants

**DOI:** 10.7759/cureus.98010

**Published:** 2025-11-28

**Authors:** Jefferson George, Indhu Poomalai, Fraser Morgan, Surya Malasani, Vivek Thakker

**Affiliations:** 1 Trauma and Orthopaedic Surgery, University Hospitals of Leicester NHS Trust, Leicester, GBR; 2 General Practice, The Countess of Chester Hospital NHS Foundation Trust, Chester, GBR

**Keywords:** diversity and inclusion, international medical graduates (imgs), medical education, online teaching, surgical education, webinar series

## Abstract

Background

The UK (United Kingdom) surgical training programme has evolved tremendously over the last decade. With the introduction of the European Working Time Directive and the lifting of the Resident Market Labour Test (RMLT) restrictions for International Medical Graduates (IMGs), there has been a shift in the dynamics of entering surgical training programmes. While these changes opened opportunities, numerous questions regarding the entry process and the nature of the training itself, such as application process, portfolio-building expectations, speciality-specific roles and day-to-day clinical responsibilities, have remained unanswered. This underscores the need for targeted educational interventions that address these practical challenges and improve preparedness for UK surgical training. This study evaluated the impact of a trainee-led, webinar-based educational programme designed to improve understanding of UK surgical training and to support aspiring surgical trainees, with a specific focus on inclusivity, accessibility and representation of IMGs.

Methods

A 7-part teaching series was created collaboratively by the West Midlands’ Foundation Trainee Surgical Society (FTSS) and the Surgical Society of International Doctors (SSID), using guidance from Health Education England and the Royal Colleges. The programme was delivered live on the MedAll platform and tailored using a pre-course needs assessment survey. Participants completed pre- and post-session questionnaires assessing confidence across four domains: understanding the surgical training pathway, application and portfolio readiness, speciality-specific roles, and management of surgical emergencies. Responses were measured using 5-point Likert scales. Quantitative data were analysed using descriptive statistics and the Wilcoxon Signed-Rank Test. Qualitative feedback underwent thematic analysis.

Results

Two hundred eighty-five attendees from 38 countries attended the teaching programme and completed the questionnaires. Statistically significant improvements were observed across all domains (*p* < 0.05), with overall confidence rising from 35% to 88%. Thematic analysis revealed four key themes: improved clarity on training structure, value of trainee-led insight, inclusivity and suggestions for session refinement.

Conclusion

This study demonstrated that a targeted and inclusive webinar series can provide measurable benefits in improving the confidence and preparedness of aspiring surgical trainees, particularly IMGs. The programme represents a scalable and sustainable model of surgical education. Future iterations may expand into national integration for supporting a diverse and well-informed UK surgical workforce.

## Introduction

Over the past decade, the landscape of surgical training in the United Kingdom (UK) has significantly changed [1]. Policy changes such as the European Working Time Directive (EWTD), the lifting of the Resident Labour Market Test (RLMT), and the inclusion of doctors on the UK Shortage Occupation List have reshaped the accessibility and structure of surgical training, particularly for International Medical Graduates (IMGs). According to the General Medical Council (GMC) Workforce Report 2022, there has been a 121% increase in the number of IMGs joining the UK workforce, highlighting the growing diversity of the National Health Service (NHS) workforce (GMC, 2022) [2]. The changes, however promising, have created a new obstacle: many IMGs continue to face an information gap regarding the structure and expectations of UK surgical training. Entry into UK surgical training remains challenging for those unfamiliar with the system. IMGs frequently report limited access to structured guidance, inadequate support for portfolio development and unfamiliarity with the speciality-specific expectations with each surgical pathway [3-4]. These barriers can result in lower application confidence, reduced competitiveness, and attrition from the surgical pipeline [5].

The need for equitable and sustainable educational initiatives in this realm is warranted, considering the diversity of the NHS workforce [6]. To the best of our knowledge, few initiatives have successfully addressed the specific needs of early career doctors from diverse and international backgrounds considering surgical careers in the UK [7-9].

To address this gap, two surgical societies undertook a successful collaboration to advance surgical education. The Surgical Society of International Doctors (SSID) worked with the West Midlands’ Foundation Trainee Surgical Society, a trainee society under the aegis of the Royal College of Surgeons of Edinburgh, to launch a global, interactive educational series. This 7-part programme was designed to provide tailored, high-impact content for junior doctors and medical students globally, covering speciality-specific insights, portfolio development, application pathways, and day-to-day realities of surgical practice in the UK.

The primary objective of this study was to evaluate the impact of a trainee-led, webinar-based educational programme on improving confidence and understanding of the UK surgical training pathway among aspiring surgical trainees, particularly IMGs. Secondary objectives included assessing participant engagement, inclusivity and relevance of the content, along with exploring the feasibility of this model as a sustainable approach to early surgical education.

## Materials and methods

Structure of the programme

The seven-part teaching series was conducted over a period of 6 months (May to October 2023) to mirror the surgical speciality preparation and application timeline (Table 1). Each session was hosted on MedAll, a widely used virtual healthcare education platform. The underlying theme of the initiative focused on equality, diversity, and inclusion in surgical education. The sessions were hosted by current UK surgical trainees from diverse backgrounds and different surgical specialities. Each session focused on speciality-specific insights, surgical portfolio development and the application process, management of common surgical emergencies, personal insights into a surgical trainee’s daily life and specific challenges of IMGs in the speciality. All webinars were designed to be highly interactive, incorporating real-time polls, live Q&A, moderated chat engagement and anonymous feedback to enhance participant involvement. This interactivity followed the principles of adult learning theory, which emphasises experience-based learning and constructivist pedagogy, which supports active engagement as a pathway to deeper understanding and practical application. The programme content was developed based on curriculum guidance from Health Education England and the Royal Colleges.

**Table 1:** Programme outline ENT: Ear, Nose & Throat Surgery; IMG: International Medical Graduate; MSRA: multi-speciality recruitment assessment

	Name of Session
Session 1	ENT Surgery - A Day in the Life & training application
Session 2	General Surgery - Training application & the IMG perspective
Session 3	Ophthalmology: A Day in the Life & preparing for the MSRA exam
Session 4	Trauma & Orthopaedic Surgery - Training Application
Session 5	Vascular Surgery - A Day in the Life & Training Application
Session 6	Maximising your portfolio for the surgical training application
Session 7	So you want to be a Urologist?

Study design

A mixed-methods, pre-post intervention design was used to evaluate the impact of the programme. Participants’ self-reported confidence and understanding were measured before and after each session. Free-text reflections and open-ended feedback were used for thematic analysis.

Participants

Participants registered for the programme via open online registration through professional networks, surgical societies and social media platforms. The programme was open to medical students and resident doctors globally with no restrictions based on geographic location, nationality, or level of training.

Ethical considerations

This study involved the collection of anonymous, voluntary feedback from participants in an educational programme. No identifiable personal or clinical data were collected, and participation had no bearing on professional assessments or advancement. According to institutional policy, the activity did not require formal ethical approval. All participants were informed that feedback was collected for evaluation and research purposes.

Data collection

Participants completed a registration form and pre- and post-session questionnaires (Tables 2-3). The registration form collected demographic information such as professional grades, country of medical school and reason for attendance. The session questionnaires employed Likert scales exploring confidence in the UK surgical training system, familiarity with the application process, awareness of challenges, perceived relevance, usefulness and engagement with the session. Open-ended white-space questions invited expectations, feedback and perceived value of the sessions.

**Table 2:** Summary of core pre-session survey questions Questions 1 to 5: Likert-Scale Confidence Questions, Questions 6 and 7: Free-text questions

	Pre-Session Questionnaire
1.	How confident are you in your understanding of the UK surgical training pathway relevant to this speciality?
2.	How confident do you feel in your preparedness to apply for surgical training?
3.	How confident are you in identifying the key components of a competitive surgical portfolio?
4.	How confident are you in your understanding of the day-to-day responsibilities of a surgical trainee in this speciality?
5.	How confident are you in your knowledge of how surgical emergencies are managed within this speciality?
6.	What do you hope to gain from attending this session? /Why are you attending this session?
7.	Do you have any specific questions for the speaker?

**Table 3:** Summary of Core Post-Session Survey Questions Questions 1 to 8: Likert-Scale Responses, Questions 9 to 12: Free-text questions

	Post-Session Questionnaire
1.	After attending this session, how confident are you in your understanding of the UK surgical training pathway?
2.	How confident do you now feel in your preparedness to pursue surgical training?
3.	How confident are you in identifying the key components of a competitive surgical portfolio?
4.	How confident are you in your understanding of the day-to-day responsibilities of a surgical trainee in this speciality?
5.	How confident are you in your knowledge of how surgical emergencies are managed within this speciality?
6.	How engaging did you find the session?
7.	The content of the session was relevant to me.
8.	How useful did you find the session?
9.	What aspects of this session most increased your confidence?
10.	What aspects did you like the most about the session?
11.	What aspects could have been better?
12.	Any final reflections/feedback on this learning experience?

Data analysis

Descriptive statistics were used to summarise participant demographics and baseline characteristics. The Wilcoxon Signed-Rank Test was employed to assess statistically significant changes in self-reported confidence before and after the sessions, with significance set at p < 0.05. Qualitative data from open-ended responses underwent thematic analysis to identify common patterns in feedback related to programme impact. Open-ended responses from post-session surveys were analysed using an inductive thematic analysis approach in the Braun and Clarke framework. The analysis was conducted manually by two independent reviewers. Initial codes were generated separately, then reviewed jointly to consolidate themes and discrepancies through discussion. While formal intercoder reliability metrics were not applied, thematic saturation was reached.

## Results

Participant characteristics

**Figure 1:**
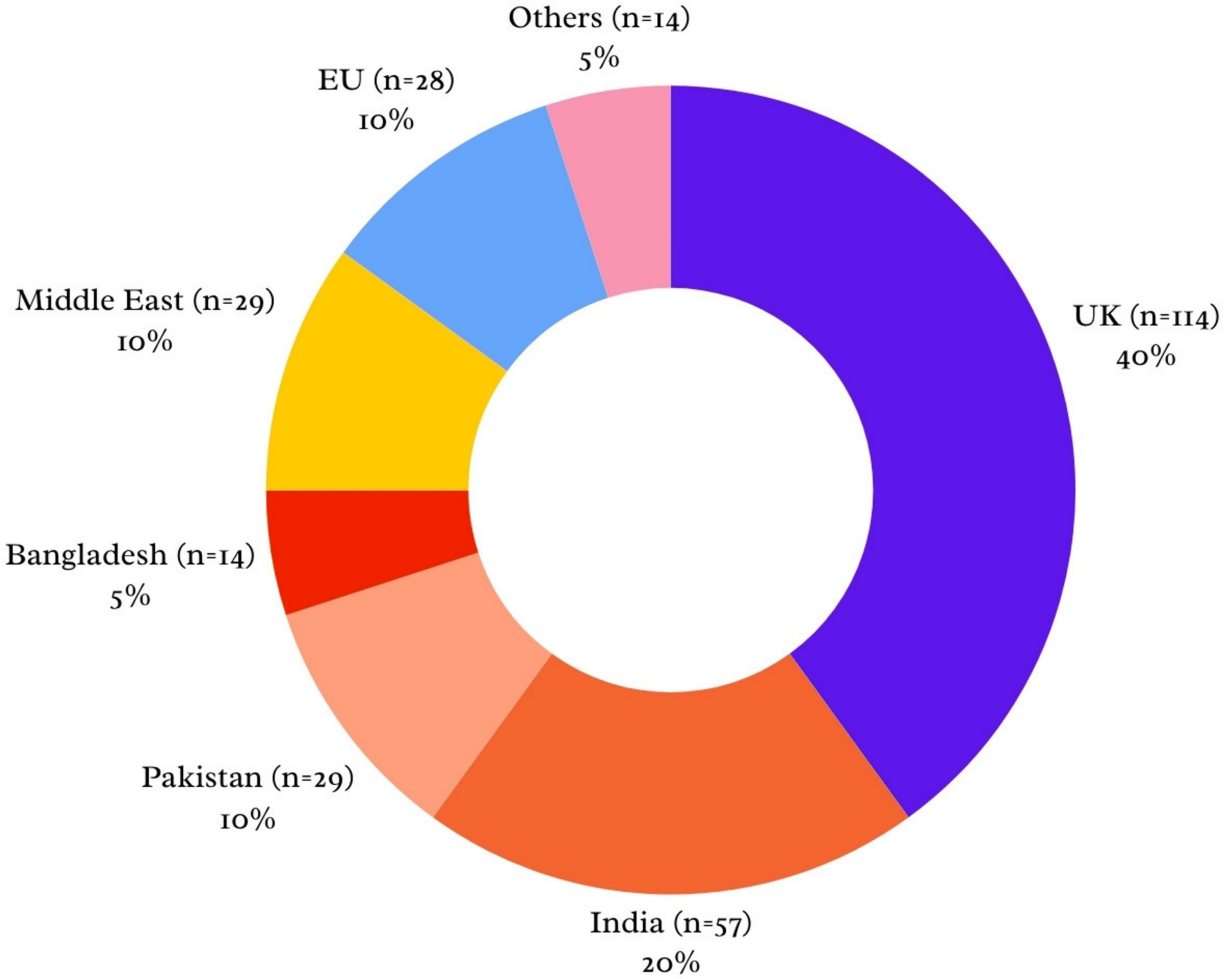
Geographic distribution of attendees EU: European Union

A total of 285 participants attended the programme and completed the associated pre- and post-session surveys. Participants represented 38 countries with a broad international distribution (Figure 1). A significant proportion (233, 82%) of attendees were IMGs who were both in the UK (140, 60%) and outside the UK (93, 40%) at the time of attendance. This international spread suggested potential variation in baseline familiarity with the UK surgical training system. The majority were either final-year medical students or resident doctors who had not entered speciality training.

**Figure 2:**
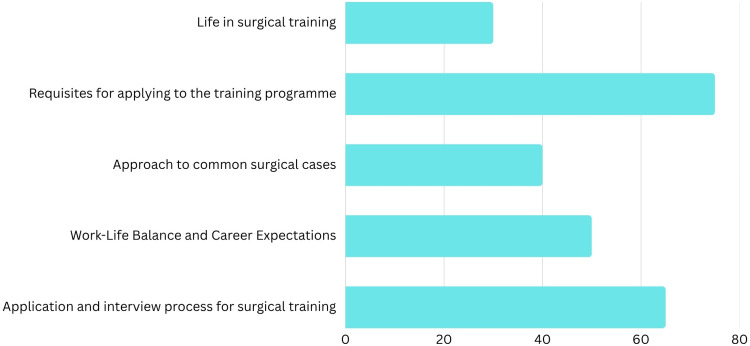
Distribution of reasons for attendance

Participants attended the sessions for different reasons. Collectively, this could be categorised into life in training, requisites for surgical training, interviews and applications, work-life balance and common surgical cases in the speciality (Figure 2). Two hundred sixty-two attendees (92%) were either interested in pursuing a surgical career in the UK or were undecided.

Quantitative results

Across all sessions, there was a statistically significant increase in participants’ self-reported confidence scores post-session compared to pre-session (Table 4).

**Table 4:** Likert-Scale data comparison pre- and post-sessions

	Pre-Session Median	Post-session Median	W Test Statistic	P-value
Understanding of the UK surgical training pathway	2.5	4.5	0	<0.001
Awareness of application and portfolio requirements	1.5	4	0	<0.001
Clarity on speciality-specific roles and responsibilities	2	4.5	0	<0.001
Familiarity with common surgical emergencies in the speciality	2.5	4.5	0	<0.001

**Figure 3:**
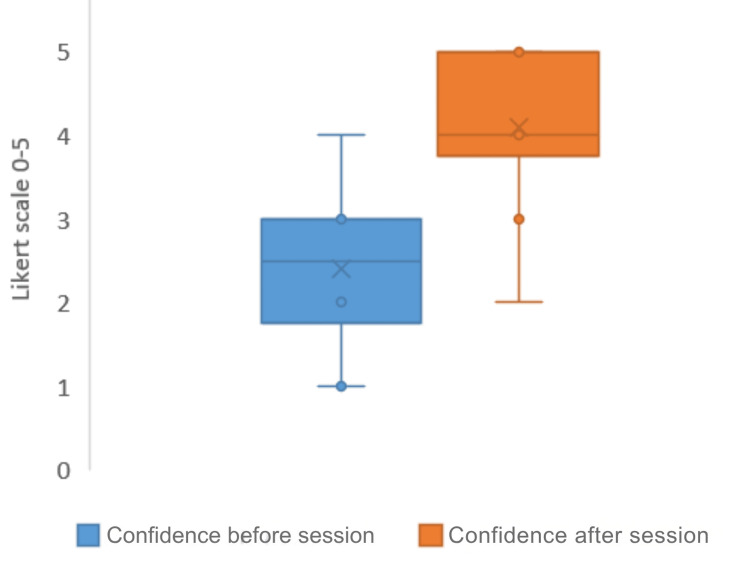
Box and whisker plot showing strong visual evidence of the session's educational impact

Participants shifted from a generally low/moderate confidence baseline to high confidence levels after the intervention across all domains (Figure 3).

Post-session confidence was positively correlated with several aspects of the teaching experience using Spearman’s rank correlation. A strong positive correlation between content relevance and post-session confidence (ρ = 0.66, p < 0.001) was seen, indicating that participants who rated the content as more relevant were significantly more likely to report higher confidence levels. Similarly, session engagement showed a moderate to strong correlation with confidence (ρ = 0.58, p < 0.001), while the perceived usefulness of the session was also strongly associated with confidence (ρ = 0.62, p < 0.001).

Qualitative results

The feedback from the white space questions was thematically analysed (Table 5). Four major themes were identified. 1) Increased Clarity Around Surgical Training Pathways, 2) Value of Insider Perspectives, 3) Inclusivity and Representation, 4) Suggestions for Improvement

**Table 5:** Thematic analysis of post-session feedback

Theme	Description	Example Quotes
Clarity on training pathways	Clearer understanding of UK surgical training entry routes and expectations	‘I understand how the application process works’, ‘The timeline makes more sense’, ‘The specific evidence required in each domain is clearer now’
Insider perspectives	Practical and honest guidance from current trainees	‘Hearing from first-hand experience in General surgery made it feel achievable’, ‘I felt like I was learning from someone who had walked the path I want to take’
Representation	Support and visibility for IMGs and women in surgery	‘As an IMG, it was good to feel that it is doable with hard work’, ‘women in surgery inspired me’
Suggestions for improvement	Feedback on session structure, content and delivery	‘More time for Q&A’, ‘the internet could be more stable’

Participants expressed that the sessions improved their understanding of the UK surgical training pathway, including entry points, portfolio requirements, and progression timelines. Imparting this clarity by those currently in the system was repeatedly emphasised, and this was the core value of the programme. The honest, experience-based insights provided the necessary authenticity to the teaching programme. This demystified career challenges and provided practical tips for national interviews and applications. Another theme that was highlighted was the programme’s focus on IMGs in surgical training. The inclusivity of the teaching programme received praise from the attendees. The unique challenges of international doctors were relatable to the attendees. Constructive feedback for refining future iterations of the programme helped maintain relevance and quality.

## Discussion

The programme demonstrated that tailored, webinar-based teachings can meaningfully increase confidence in navigating the UK surgical training system among medical students and resident doctors, particularly IMGs unfamiliar with the pathway. Analysis of the feedback also showed that participants felt included and motivated. Apart from the mere transfer of knowledge, it helped build identity and readiness in a changing landscape. Previous studies investigating the challenges faced by IMGs have highlighted a significant lack of guidance in navigating training applications in the NHS [3,9]. This programme directly addressed this gap by offering first-hand information in a flexible format. Peer-led teaching has also been reported to be a superior learning method in conveying essential information and boosting confidence [10]. Peer-led guidance also permits a smoother transition to practice for new doctors [11]. The results of our project align with this existing evidence as there was a correlation between post-session confidence and participants’ ratings of session relevance, engagement and usefulness. While strong correlations were observed, it is worth mentioning that no causal inferences can be drawn owing to the design of this project. An element of constructivism was elucidated as attendees felt motivated when learning was closely related to their life situation, professional role and goals [12]. Another notable outcome was inclusivity. Participants who were IMGs highlighted the importance of hearing from role models who shared their background. This aligns with emerging literature on the role of representation in surgical recruitment and its impact on retention and confidence [13]. By intentionally incorporating speakers from diverse backgrounds, the programme fulfilled both educational and equity functions, an increasingly important goal in modern surgical workforce planning [14].

**Figure 4:**
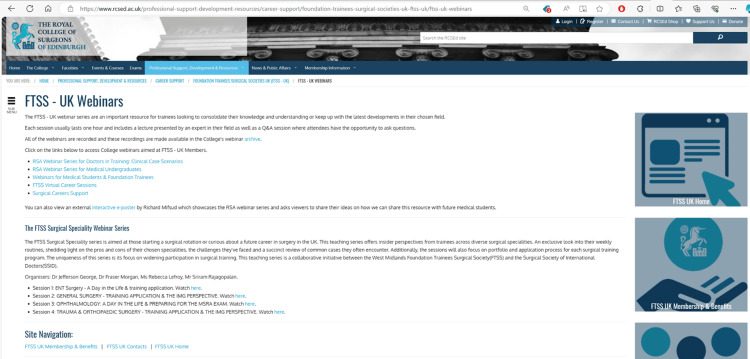
Teaching series as seen on the Royal College Website

**Figure 5:**
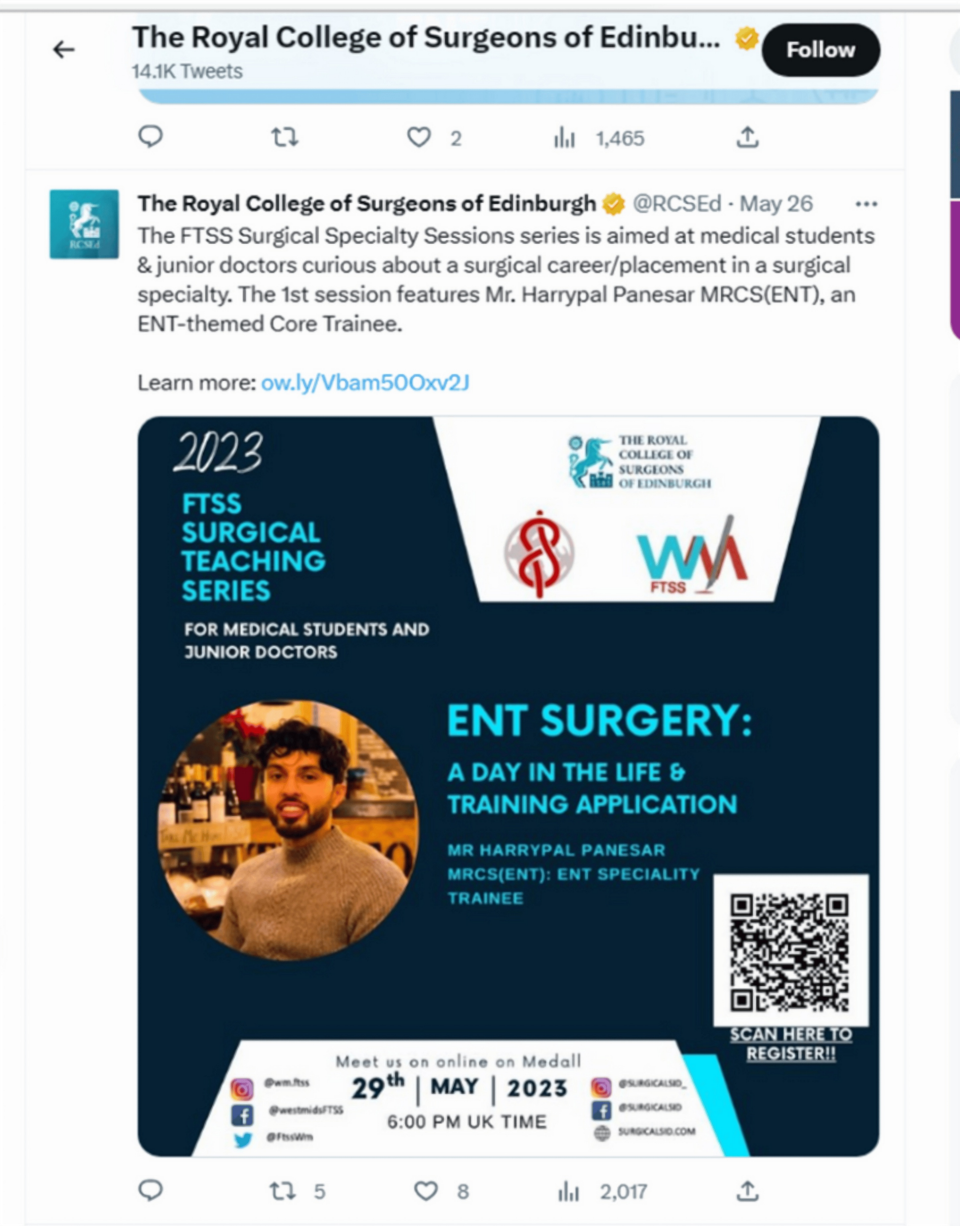
Teaching series advertised by the Royal Colleges on social media

The study and the educational initiative it evaluates have several strengths. With 285 attendees from 38 countries, the programme demonstrated a diverse surgical audience. By using quantitative and qualitative data to evaluate the impact, the study achieved triangulation of evidence through a mixed-methods approach. A pre-programme needs assessment helped tailor each session to the audience. Unlike other teaching initiatives, the sessions intentionally brought in speakers who were IMGs. This empowered the majority of the attendees. Delivering the sessions through a platform that was live, interactive and cost-effective ensured the initiative to be replicable. The sessions are now permanently hosted on the Foundation Trainee Surgical Society (FTSS) page of the Royal College of Surgeons of Edinburgh (RCSEd), signifying its ongoing visibility, credibility and access for future learners (Figures 4-5) [15].

Despite promising results, several limitations should be considered when interpreting the findings. Self-reported Likert scales are inherently subjective and may not directly correlate with actual knowledge acquisition. Although during each session, anonymous polls were used as spontaneous knowledge checks, future iterations would benefit from incorporating objective knowledge checks to provide a more comprehensive evaluation of educational impact. The evaluation captured only short-term changes in confidence. Without follow-up beyond the immediate post-session period, it is unclear whether improvements were retained over time. Another limitation is the heterogeneity of the participants who were at varying stages of training. This variability may have influenced baseline confidence levels and perceived gains, potentially confounding results. Given the voluntary nature of participation, the results may be subject to selection bias, with more motivated individuals being more likely to engage with the programme and its evaluation. As a single-arm educational intervention, the study lacked a control or comparator group. About 22 participants (7.7%) reported technical difficulties with the delivery platform, including connection drops and limited functionality in certain regions. Although the programme had international reach, its content was specifically tailored to the UK surgical training pathway. The sessions may thus not be generalisable to other surgical education systems [14]. However, the underlying structure of the initiative offers a transferable framework that can be adapted to different national training pathways to support early career doctors.

Our initiative showed that tailored, peer-led educational interventions can significantly improve the preparedness of aspiring surgical trainees. The results call for further educational initiatives that respect the diversity in surgical training. Given the rising proportion of IMGs in the UK workforce, targeted preparatory resources are essential to equitable recruitment and performance [6,16]. IMGs often feel ill-prepared and under-informed about NHS expectations, training routes, and clinical culture upon arrival in the UK [17]. The success of the initiative shows the scalability of a cost-effective endeavour to reduce such disparities in surgical education and career progression [18].

## Conclusions

Future studies should look at whether educational interventions similar to ours translate to real-world achievement in milestones such as successful training applications, higher interview or portfolio scores or improved scores in the multi-speciality recruitment assessment (MSRA) exams. One feasible approach would be to implement a closed-loop follow-up survey distributed to participants at the end of the national recruitment cycle to assess impact. Our educational initiative can be mirrored to include a wider range of specialities, alternative routes to training and non-technical skills for surgery, such as leadership and research. Future efforts should also focus on collaborative scaling through strategic partnerships with key organisations such as the GMC, NHS England and Association of Surgeons in Training.

By combining evidence-informed content with trainee-led delivery, this study shows a scalable and sustainable initiative of education for widening access to surgical careers in the UK. The project showed measurable benefit through a mixed-methods evaluation. With further development, there is significant potential for integration of this model into national induction and onboarding programmes, ultimately supporting an equitable and well-prepared surgical workforce.
